# Methyl 2-(4-hy­droxy-1-methyl-2-oxo-1,2-dihydro­quinolin-3-yl)acetate

**DOI:** 10.1107/S1600536810046453

**Published:** 2010-11-17

**Authors:** Svitlana V. Shishkina, Oleg V. Shishkin, Igor V. Ukrainets, Elena V. Mospanova

**Affiliations:** a STC "Institute for Single Crystals", National Academy of Sciences of Ukraine, 60 Lenina ave., Kharkiv 61001, Ukraine; b National University of Pharmacy, 4 Blyukhera ave., Kharkiv 61002, Ukraine

## Abstract

In the title compound, C_13_H_13_NO_4_, the bicyclic quinolone fragment and the ester group are approximately orthogonal, making a dihedral angle of 83.3 (2)° and an intramolecular C—H⋯O interaction occurs. In the crystal, inter­molecular O—H⋯O hydrogen bonding generates a zigzag chain along the *c* axis.

## Related literature

Esters of 4-hy­droxy-2-oxo-1,2-dihydro­quinolin-3-acetic acids reveal appreciable biological activity, see: Ukrainets *et al.* (2010 ▶). For a related structure, see: Ukrainets *et al.* (2009 ▶). For van der Waals radii, see: Zefirov (1997 ▶). For reference bond lengths, see: Bürgi & Dunitz (1994 ▶).
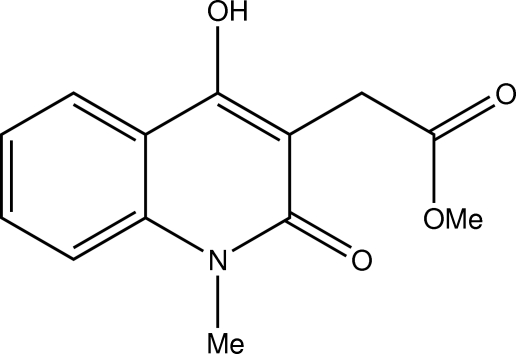


## Experimental

### 

#### Crystal data

C_13_H_13_NO_4_
*M* *_r_* = 247.24
Monoclinic, 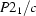

*a* = 9.0792 (6) Å
*b* = 11.4904 (6) Å
*c* = 11.4071 (7) Å
β = 105.272 (7)°
*V* = 1148.00 (12) Å^3^
*Z* = 4
Mo *K*α radiation
μ = 0.11 mm^−1^
*T* = 293 K
0.20 × 0.10 × 0.10 mm

#### Data collection

Oxford Xcalibur3 diffractometer
11774 measured reflections
3295 independent reflections
1454 reflections with *I* > 2σ(*I*)
*R*_int_ = 0.031

#### Refinement

*R*[*F* ^2^ > 2σ(*F* ^2^)] = 0.035
*wR*(*F* ^2^) = 0.082
*S* = 0.72
3295 reflections
169 parameters
H atoms treated by a mixture of independent and constrained refinement
Δρ_max_ = 0.14 e Å^−3^
Δρ_min_ = −0.14 e Å^−3^

### 

Data collection: *CrysAlis CCD* (Oxford Diffraction, 2005 ▶); cell refinement: *CrysAlis RED*; data reduction: *CrysAlis RED*; program(s) used to solve structure: *SHELXTL* (Sheldrick, 2008 ▶); program(s) used to refine structure: *SHELXTL*; molecular graphics: *XP* (Siemens, 1998 ▶); software used to prepare material for publication: *SHELXTL*.

## Supplementary Material

Crystal structure: contains datablocks I, global. DOI: 10.1107/S1600536810046453/kp2282sup1.cif

Structure factors: contains datablocks I. DOI: 10.1107/S1600536810046453/kp2282Isup2.hkl

Additional supplementary materials:  crystallographic information; 3D view; checkCIF report

## Figures and Tables

**Table 1:** Hydrogen-bond geometry (Å, °)

*D*—H⋯*A*	*D*—H	H⋯*A*	*D*⋯*A*	*D*—H⋯*A*
O2—H2*O*⋯O1^i^	0.921 (17)	1.760 (17)	2.6456 (12)	160.2 (15)
C10—H10*A*⋯O1^i^	0.97	2.48	3.3335 (16)	147

Symmetry code: (i) 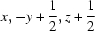.

**Fig. 1.**
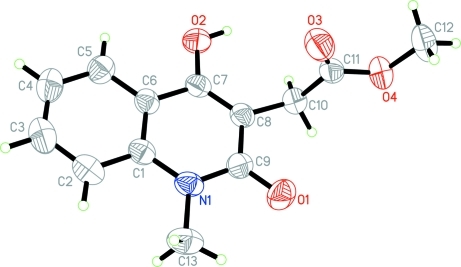
View of the title compound with atomic membering. All atoms are shown with displacement ellipsoids drawn at the 50% probability level.

**Fig. 2.**
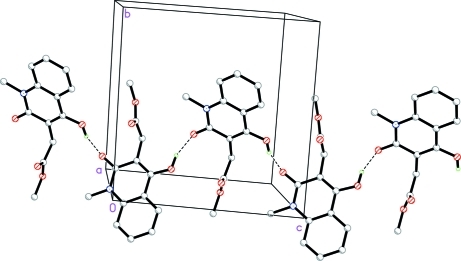
Crystal packing of the title molecules. The hydrogen bonds are shown by dashed lines.

## supplementary crystallographic information

### Comment

Esters of 4-hydroxy-2-oxo-1,2-dihydroquinolin-3-acetic acids reveal appreciable biological activity (Ukrainets *et al.*, 2010). It is interesting that the ethyl ester possesses stronger anti-inflammory activity than methyl ester. On the contrary, the methyl ester has pronounced analgetic activity. In this paper we report the molecular and crystal structure of the (4-hydroxy-1-methyl-2-oxo-1,2- dihydroquinolin-3-yl)-acetic acid methyl ester (I) (Fig. 1) with a comparative analysis with previously studied ethyl analogue (II) (Ukrainets *et al.*, 2009). In contrast to II the bicyclic fragment of I is not strictly planar (the C1—N1—C9—C8 torsion angle is -5.8 (2) °). The planar ester group at the C10 atom is orthogonal to the plane of quinoline ring (the C7—C8—C10—C11 torsion angle is 93.9 (1) °) and the C8—C10—C11—O3 torsion angle is -19.7 (2) °. The C9—O1 bond (1.251 (1) Å) is elongated comparing with its mean value (1.210 Å; Bürgi & Dunitz, 1994) owing to the formation of the intermolecular hydrogen bond O2—H2O···O1 (Table 1). The presence of this hydrogen bond determines the orientation of the hydrogen atom of hydroxy group despite of repulsion with one of hydrogen atoms of neighbouring methylene group: the H10*a*···H2O distance is 2.09 Å [the van der Waals radii sum is 2.34 Å (Zefirov, 1997)]. In the crystal packing the molecules are connected by the O2—H2O···O1 intermolecular hydrogen bond into a zigzag chain along the [0 0 1] direction (Table 1, Fig. 2). Neighbouring chains are connected by the C-H···π interactions between the methyl group at N1-pyridyl atom and C1···C6 aromatic ring [-x, 1-y, 1-z; H···Cg distance (Cg is centre of aromatic ring) is 3.28 Å, C-H···Cg bond angle is 125 °].

### Experimental

(4-hydroxy-1-methyl-2-oxo-1,2-dihydroquinolin-3-yl)-acetic acid is synthesised using the published method (Ukrainets *et al.*, 2010). Yield 96%; m.p. 452-454 K.

### Refinement

All hydrogen atoms were located from electron density difference maps and were refined in the riding mode approximation with *U*_iso_ constrained to be 1.5 times *U*_eq_ of the carrier atom for the methyl group and 1.2 times *U*_eq_ of the carrier atom for the other atoms. The hydrogen atom of the hydroxyl group was refined in an isotropic mode.

### Crystal data

**Table:**

C_13_H_13_NO_4_	*F*(000) = 520
*M**_r_* = 247.24	*D*_x_ = 1.431 Mg m^−^^3^
Monoclinic, *P*2_1_/*c*	Mo *K*α radiation, λ = 0.71073 Å
*a* = 9.0792 (6) Å	Cell parameters from 3049 reflections
*b* = 11.4904 (6) Å	θ = 2.9–32.1°
*c* = 11.4071 (7) Å	µ = 0.11 mm^−^^1^
β = 105.272 (7)°	*T* = 293 K
*V* = 1148.00 (12) Å^3^	Block, colourless
*Z* = 4	0.20 × 0.10 × 0.10 mm

### Data collection

**Table:**

Oxford Xcalibur3 diffractometer	1454 reflections with *I* > 2σ(*I*)
Radiation source: Enhance (Mo) X-ray Source	*R*_int_ = 0.031
graphite	θ_max_ = 30.0°, θ_min_ = 2.9°
Detector resolution: 16.1827 pixels mm^-1^	*h* = −12→12
ω scans	*k* = −15→16
11774 measured reflections	*l* = −16→16
3295 independent reflections	

### Refinement

**Table:**

Refinement on *F*^2^	Primary atom site location: structure-invariant direct methods
Least-squares matrix: full	Secondary atom site location: difference Fourier map
*R*[*F*^2^ > 2σ(*F*^2^)] = 0.035	Hydrogen site location: difference Fourier map
*wR*(*F*^2^) = 0.082	H atoms treated by a mixture of independent and constrained refinement
*S* = 0.72	*w* = 1/[σ^2^(*F*_o_^2^) + (0.044*P*)^2^] where *P* = (*F*_o_^2^ + 2*F*_c_^2^)/3
3295 reflections	(Δ/σ)_max_ < 0.001
169 parameters	Δρ_max_ = 0.14 e Å^−^^3^
0 restraints	Δρ_min_ = −0.14 e Å^−^^3^

### Special details

**Table:**

Geometry. All e.s.d.'s (except the e.s.d. in the dihedral angle between two l.s. planes) are estimated using the full covariance matrix. The cell e.s.d.'s are taken into account individually in the estimation of e.s.d.'s in distances, angles and torsion angles; correlations between e.s.d.'s in cell parameters are only used when they are defined by crystal symmetry. An approximate (isotropic) treatment of cell e.s.d.'s is used for estimating e.s.d.'s involving l.s. planes.
Refinement. Refinement of *F*^2^ against ALL reflections. The weighted *R*-factor *wR* and goodness of fit *S* are based on *F*^2^, conventional *R*-factors *R* are based on *F*, with *F* set to zero for negative *F*^2^. The threshold expression of *F*^2^ > σ(*F*^2^) is used only for calculating *R*-factors(gt) *etc*. and is not relevant to the choice of reflections for refinement. *R*-factors based on *F*^2^ are statistically about twice as large as those based on *F*, and *R*- factors based on ALL data will be even larger.

### Fractional atomic coordinates and isotropic or equivalent isotropic displacement parameters (Å^2^)

**Table:**

	*x*	*y*	*z*	*U*_iso_*/*U*_eq_	
N1	0.27207 (11)	0.49052 (8)	0.49471 (8)	0.0442 (3)	
O1	0.39749 (11)	0.33292 (7)	0.44953 (8)	0.0582 (3)	
O2	0.26304 (11)	0.34913 (8)	0.82386 (8)	0.0565 (3)	
H2O	0.3143 (18)	0.2811 (15)	0.8511 (15)	0.100 (6)*	
O3	0.19057 (12)	0.11214 (7)	0.58965 (9)	0.0613 (3)	
O4	0.41580 (10)	0.02233 (7)	0.61834 (8)	0.0576 (3)	
C1	0.19562 (14)	0.54717 (10)	0.56935 (11)	0.0412 (3)	
C2	0.11931 (15)	0.65295 (10)	0.53520 (13)	0.0538 (4)	
H2	0.1204	0.6877	0.4618	0.065*	
C3	0.04276 (16)	0.70527 (12)	0.61044 (14)	0.0615 (4)	
H3	−0.0073	0.7755	0.5873	0.074*	
C4	0.03911 (16)	0.65520 (11)	0.71953 (14)	0.0591 (4)	
H4	−0.0132	0.6916	0.7693	0.071*	
C5	0.11239 (14)	0.55205 (11)	0.75432 (12)	0.0500 (3)	
H5	0.1093	0.5183	0.8277	0.060*	
C6	0.19244 (13)	0.49640 (9)	0.68027 (10)	0.0404 (3)	
C7	0.27042 (13)	0.38819 (10)	0.71437 (10)	0.0411 (3)	
C8	0.34114 (14)	0.33278 (9)	0.63922 (11)	0.0416 (3)	
C9	0.33855 (14)	0.38309 (10)	0.52337 (11)	0.0433 (3)	
C10	0.42339 (14)	0.21863 (10)	0.66808 (12)	0.0487 (3)	
H10B	0.5113	0.2192	0.6348	0.058*	
H10A	0.4607	0.2112	0.7556	0.058*	
C11	0.32703 (16)	0.11471 (10)	0.61955 (11)	0.0439 (3)	
C12	0.33896 (18)	−0.08538 (12)	0.57708 (14)	0.0681 (4)	
H12C	0.2641	−0.1004	0.6209	0.102*	
H12B	0.4120	−0.1477	0.5910	0.102*	
H12A	0.2895	−0.0800	0.4918	0.102*	
C13	0.27996 (17)	0.54417 (12)	0.38000 (11)	0.0600 (4)	
H13C	0.3199	0.6217	0.3953	0.090*	
H13B	0.1795	0.5472	0.3254	0.090*	
H13A	0.3456	0.4988	0.3442	0.090*	

### Atomic displacement parameters (Å^2^)

**Table:**

	*U*^11^	*U*^22^	*U*^33^	*U*^12^	*U*^13^	*U*^23^
N1	0.0566 (7)	0.0417 (6)	0.0338 (6)	−0.0103 (5)	0.0112 (5)	−0.0002 (4)
O1	0.0705 (6)	0.0588 (6)	0.0493 (6)	−0.0083 (5)	0.0230 (5)	−0.0177 (4)
O2	0.0725 (7)	0.0558 (6)	0.0474 (6)	0.0058 (5)	0.0269 (5)	0.0123 (5)
O3	0.0581 (6)	0.0474 (6)	0.0775 (7)	−0.0025 (5)	0.0159 (5)	−0.0014 (4)
O4	0.0650 (6)	0.0387 (5)	0.0674 (6)	0.0057 (4)	0.0144 (5)	−0.0046 (4)
C1	0.0428 (7)	0.0371 (7)	0.0410 (7)	−0.0088 (5)	0.0064 (5)	−0.0028 (5)
C2	0.0580 (9)	0.0469 (8)	0.0511 (9)	−0.0068 (7)	0.0049 (7)	0.0062 (6)
C3	0.0560 (9)	0.0444 (8)	0.0781 (11)	0.0047 (7)	0.0071 (8)	−0.0007 (7)
C4	0.0554 (9)	0.0539 (9)	0.0672 (10)	0.0045 (7)	0.0150 (7)	−0.0136 (7)
C5	0.0506 (8)	0.0524 (8)	0.0467 (8)	−0.0019 (6)	0.0125 (6)	−0.0069 (6)
C6	0.0444 (7)	0.0354 (6)	0.0403 (7)	−0.0057 (5)	0.0090 (5)	−0.0048 (5)
C7	0.0478 (7)	0.0382 (7)	0.0375 (7)	−0.0080 (6)	0.0117 (6)	−0.0002 (5)
C8	0.0464 (7)	0.0355 (6)	0.0435 (7)	−0.0070 (5)	0.0127 (6)	−0.0027 (5)
C9	0.0468 (7)	0.0417 (7)	0.0423 (8)	−0.0117 (6)	0.0131 (6)	−0.0099 (5)
C10	0.0529 (8)	0.0414 (7)	0.0537 (8)	0.0001 (6)	0.0174 (6)	−0.0016 (6)
C11	0.0577 (9)	0.0385 (7)	0.0377 (7)	0.0003 (7)	0.0164 (6)	0.0019 (5)
C12	0.0916 (11)	0.0384 (8)	0.0681 (10)	0.0022 (7)	0.0100 (8)	−0.0086 (6)
C13	0.0731 (10)	0.0644 (9)	0.0426 (8)	−0.0146 (7)	0.0152 (7)	0.0062 (6)

### Geometric parameters (Å, °)

**Table:**

N1—C9	1.3751 (15)	C4—H4	0.9300
N1—C1	1.3936 (15)	C5—C6	1.4049 (16)
N1—C13	1.4652 (15)	C5—H5	0.9300
O1—C9	1.2512 (14)	C6—C7	1.4330 (16)
O2—C7	1.3454 (14)	C7—C8	1.3574 (16)
O2—H2O	0.921 (17)	C8—C9	1.4370 (17)
O3—C11	1.1957 (14)	C8—C10	1.5023 (15)
O4—C11	1.3351 (14)	C10—C11	1.4975 (17)
O4—C12	1.4378 (15)	C10—H10B	0.9700
C1—C6	1.4005 (16)	C10—H10A	0.9700
C1—C2	1.4025 (17)	C12—H12C	0.9600
C2—C3	1.3766 (19)	C12—H12B	0.9600
C2—H2	0.9300	C12—H12A	0.9600
C3—C4	1.379 (2)	C13—H13C	0.9600
C3—H3	0.9300	C13—H13B	0.9600
C4—C5	1.3660 (17)	C13—H13A	0.9600
			
C9—N1—C1	122.15 (10)	C7—C8—C10	124.17 (11)
C9—N1—C13	117.93 (11)	C9—C8—C10	116.07 (11)
C1—N1—C13	119.90 (11)	O1—C9—N1	119.49 (11)
C7—O2—H2O	116.9 (10)	O1—C9—C8	121.83 (12)
C11—O4—C12	116.46 (10)	N1—C9—C8	118.66 (11)
N1—C1—C6	119.31 (11)	C11—C10—C8	114.01 (10)
N1—C1—C2	121.62 (11)	C11—C10—H10B	108.8
C6—C1—C2	119.06 (12)	C8—C10—H10B	108.8
C3—C2—C1	119.92 (13)	C11—C10—H10A	108.8
C3—C2—H2	120.0	C8—C10—H10A	108.8
C1—C2—H2	120.0	H10B—C10—H10A	107.6
C2—C3—C4	121.09 (13)	O3—C11—O4	124.09 (11)
C2—C3—H3	119.5	O3—C11—C10	125.86 (11)
C4—C3—H3	119.5	O4—C11—C10	110.01 (11)
C5—C4—C3	119.90 (13)	O4—C12—H12C	109.5
C5—C4—H4	120.1	O4—C12—H12B	109.5
C3—C4—H4	120.1	H12C—C12—H12B	109.5
C4—C5—C6	120.65 (13)	O4—C12—H12A	109.5
C4—C5—H5	119.7	H12C—C12—H12A	109.5
C6—C5—H5	119.7	H12B—C12—H12A	109.5
C1—C6—C5	119.38 (11)	N1—C13—H13C	109.5
C1—C6—C7	118.71 (11)	N1—C13—H13B	109.5
C5—C6—C7	121.91 (11)	H13C—C13—H13B	109.5
O2—C7—C8	125.26 (11)	N1—C13—H13A	109.5
O2—C7—C6	113.55 (10)	H13C—C13—H13A	109.5
C8—C7—C6	121.18 (11)	H13B—C13—H13A	109.5
C7—C8—C9	119.75 (11)		
			
C9—N1—C1—C6	3.30 (16)	O2—C7—C8—C9	178.97 (10)
C13—N1—C1—C6	−178.30 (10)	C6—C7—C8—C9	0.14 (17)
C9—N1—C1—C2	−175.55 (11)	O2—C7—C8—C10	−0.41 (19)
C13—N1—C1—C2	2.85 (17)	C6—C7—C8—C10	−179.24 (10)
N1—C1—C2—C3	178.85 (11)	C1—N1—C9—O1	175.57 (11)
C6—C1—C2—C3	−0.01 (17)	C13—N1—C9—O1	−2.86 (16)
C1—C2—C3—C4	−0.2 (2)	C1—N1—C9—C8	−5.80 (16)
C2—C3—C4—C5	0.0 (2)	C13—N1—C9—C8	175.77 (11)
C3—C4—C5—C6	0.3 (2)	C7—C8—C9—O1	−177.38 (11)
N1—C1—C6—C5	−178.52 (11)	C10—C8—C9—O1	2.05 (17)
C2—C1—C6—C5	0.36 (16)	C7—C8—C9—N1	4.03 (17)
N1—C1—C6—C7	0.99 (15)	C10—C8—C9—N1	−176.54 (10)
C2—C1—C6—C7	179.87 (11)	C7—C8—C10—C11	93.90 (14)
C4—C5—C6—C1	−0.53 (18)	C9—C8—C10—C11	−85.50 (13)
C4—C5—C6—C7	179.98 (11)	C12—O4—C11—O3	0.54 (18)
C1—C6—C7—O2	178.39 (10)	C12—O4—C11—C10	178.27 (11)
C5—C6—C7—O2	−2.12 (16)	C8—C10—C11—O3	−19.67 (18)
C1—C6—C7—C8	−2.65 (16)	C8—C10—C11—O4	162.65 (10)
C5—C6—C7—C8	176.84 (12)		

### Hydrogen-bond geometry (Å, °)

**Table:**

*D*—H···*A*	*D*—H	H···*A*	*D*···*A*	*D*—H···*A*
O2—H2O···O1^i^	0.921 (17)	1.760 (17)	2.6456 (12)	160.2 (15)
C10—H10A···O1^i^	0.97	2.48	3.3335 (16)	147
C5—H5···O2	0.93	2.40	2.7138 (16)	100

Symmetry codes: (i) *x*, −*y*+1/2, *z*+1/2.
